# Rapid RASER MRI

**DOI:** 10.1002/anie.202525699

**Published:** 2026-01-28

**Authors:** Sören Lehmkuhl, Simon Fleischer, Jing Yang, Eduard Y. Chekmenev, Thomas Theis, Stephan Appelt, Jan G. Korvink, Mazin Jouda

**Affiliations:** ^1^ Institute of Microstructure Technology Karlsruhe Institute of Technology Eggenstein‐Leopoldshafen 76344 Karlsruhe Germany; ^2^ Department of Chemistry Integrative Biosciences Karmanos Cancer Institute Wayne State University Detroit Michigan 48202 USA; ^3^ Department of Chemistry Department of Physics Comparative Medicine Institute North Carolina State University Raleigh North Carolina 27695–8204, 513 USA; ^4^ Institute of Technical and Macromolecular Chemistry RWTH Aachen University 52056 Aachen Germany; ^5^ Peter Grünberg Institute (PGI‐4) Integrated Computing Architectures (ICA) Forschungszentrum Jülich GmbH 52425 Jülich Germany

**Keywords:** Hyperpolarization, MRI, NMR spectroscopy, Parahydrogen, RASER

## Abstract

Conventional Magnetic Resonance Imaging (MRI) relies on high‐power Radio‐Frequency (RF) pulses to excite nuclear spins and in turn generate NMR signals. These pulses require large high‐power RF‐amplifiers and cause heat deposition in the tissue, which must be minimized for safety, presenting a growing problem when moving toward ever‐higher field MRI. An alternative to RF‐pulse excitation is self‐excitation of nuclear spins using Radiofrequency Amplification by Stimulated Emission of Radiation (RASER), where the nuclear spins undergo spontaneous transition, without RF excitation, from an over‐populated state to a ground state. Here, the feasibility of recording rapid proton RASER MRI images of pyrazine at low concentration (120 mM) with large matrix (128x128 pixels) in as little as 78 ms is demonstrated at 500 MHz (11.7 T). We also recorded a time‐series of images using a single bolus hyperpolarized pyrazine highlighting the feasibility of dynamic tracking. The demonstrated approach allows recording MRI scans without transmit‐receive electronics of the MRI scanner, which is highly desirable for portable MRI as well as the emerging field of hyperpolarized MRI using, e.g., HP protons, ^129^Xe gas or HP ^13^C labeled biomolecules as molecular tracers and imaging agents.

## Graphical abstract

Rapid RASER MRI allows for image acquisition without RF excitation. Instead, a population inversion on the nuclear spins is generated by SABRE hyperpolarization, which allows for self‐excitation through the RASER approach. When the desired transverse magnetization is built‐up, conventional EPI encoding is triggered to record the image.

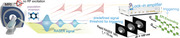

Magnetic Resonance (MR) technology is indispensable for chemical analysis, material science, and medicine. To further increase the versatility of NMR and MRI technologies, there has always been a desire for higher sensitivity to molecules at low concentrations. One emerging technology to boost NMR and MRI sensitivity is hyperpolarization. Unlike conventional MR technologies, which rely on a small population difference of nuclear spin energy levels, hyperpolarization creates large (near‐unity) population differences between nuclear‐spin‐energy levels. Signal enhancements up to seven orders of magnitude have been reported.^[^ ^1^ ^]^ As a result, it becomes possible to detect low‐concentration analytes, which have emerged as contrast media for a next‐generation of molecular imaging. For example, hyperpolarized (HP) ^1^H enhances chemical analysis,^[^ ^2^, ^3^, ^4^ ^]^ HP ^129^Xe is FDA‐approved for functional pulmonary imaging,^[^ ^5^ ^]^ and HP [^13^C]pyruvate is employed in over 50 clinical trials for imaging of metabolic flux and reporting on aberrant metabolism in cancer or other metabolic diseases.^[^ ^6^ ^]^

HP contrast agents often rely on heteronuclei (*e.g*., ^13^C and ^129^Xe) because of extended T_1_ times. However, heteronuclei resonate at much lower frequencies than those of protons in tissue, which are conventionally detected in clinical MRI scanners. Since the radio frequency (RF) chain of clinical MRI scanners is narrowband in the frequency domain, scanning of HP heteronuclei is not readily possible on clinical MRI scanners.

Conventional MRI scanners use RF pulses to excite the nuclear spins into a coherence between the ground state and an excited state, followed by acquisition of the MR signal generated by the nuclear spin ensemble precessing around the main magnetic field. The alternative to external RF excitation is self‐excitation of the nuclear spins, which can be achieved by creating a HP excited state, establishing a population inversion. This population inversion can produce RASER activity above a given threshold,^[^ ^7^, ^8^, ^9^ ^]^ akin to its big brother the LASER, which requires population inversions of electronic excited states instead of nuclear spin states used by the RASER.

In this work, we employ SABRE (Signal Amplification by Reversible Exchange)^[^ ^10^, ^11^ ^]^ that relies on simultaneous chemical exchange of parahydrogen (source of polarization) and target molecule (pyrazine employed in this work). SABRE hyperpolarization creates an inverted HP proton spin state on pyrazine as depicted in Scheme 1 (we note that the inverted state, providing RASER self‐excitation, can also be generated by other hyperpolarization techniques, including Dynamic Nuclear Polarization (DNP)^[^ ^12^, ^13^ ^]^ Spin Exchange Optical Pumping) SEOP,^[^ ^14^, ^15^ ^]^ or other Parahydrogen Induced Polarization (PHIP) methods ^[^ ^16^, ^17^ ^]^). For the present case of SABRE hyperpolarization,^[^ ^11^ ^]^ spin order from parahydrogen is transferred to the target substrate via a temporarily formed *J*‐coupling network at a transition metal catalyst. One particularly appealing feature of SABRE is that the hyperpolarization can be replenished continuously as both hydrogen and the target substrate reversibly exchange at the catalyst (Scheme 1).

**Scheme 1:**
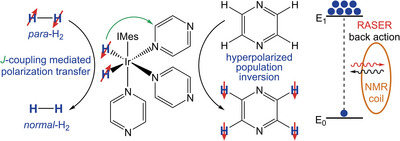
SABRE hyperpolarization. Spin order is transferred from parahydrogen through the temporarily formed *J*‐coupling network on the transition metal catalyst to build up polarization on the target molecule, pyrazine. Reversible exchange allows coordination of parahydrogen and build‐up of high degrees of population inversion at 6.5 mT on the uncoordinated substrate. The interaction of the sample with the coil leads to emission of RASER signal because of the back action of the induced current in the NMR coil on the spins.

For the RASER imaging experiments, we utilized a workflow that allowed rapid image detection using the RASER approach for excitation (Figure 1). Specifically, each sample was pressurized with 6 bar parahydrogen, shaken for 20 s in the stray field of the 500 MHz NMR, and exposed to a field of 6.5 mT for 10 s to provide efficient SABRE hyperpolarization before transfer into the NMR magnet for detection (Figure 1a). Following the interaction of the inverted HP state with the NMR sensing RF circuit, XY magnetization (i.e., a RASER signal) emerges spontaneously, without the application of any RF‐excitation pulse. For such RASER experiments, the coil is connected to a lock‐in amplifier that monitors the signal and sets a trigger for image encoding. As soon as the RASER‐induced signal reaches a predefined voltage threshold, gradient‐based echo‐planar image (EPI) encoding is triggered.

**Figure 1:**
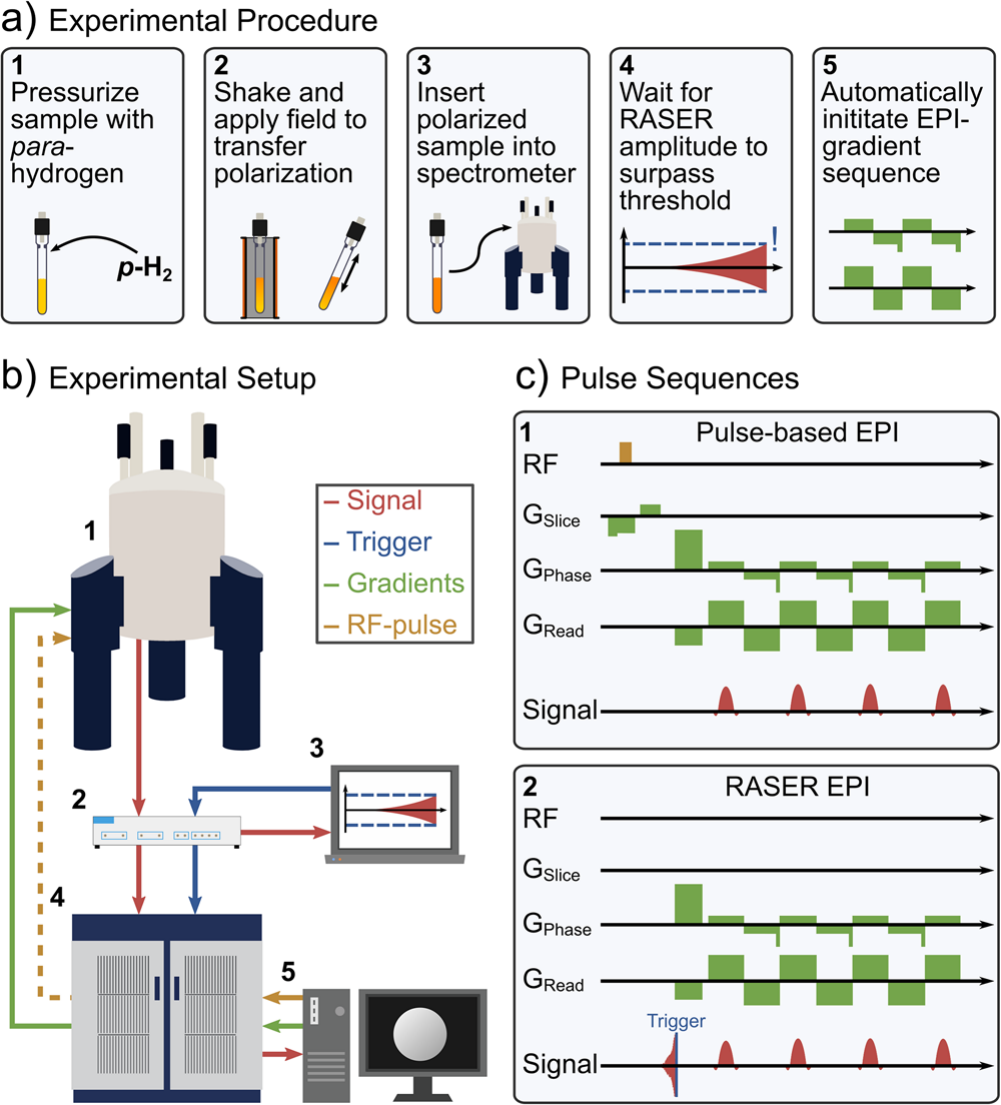
Rapid RASER MRI. a): Experimental procedure: The sample is pressurized with parahydrogen (1), SABRE hyperpolarized (2), and inserted into the NMR magnet (3). The RASER starts spontaneously (4). Once a pre‐set signal amplitude is detected, EPI acquisition is triggered (5). b) Experimental setup: Once the pre‐set signal amplitude is reached and detected on the laptop (3, red), a trigger is sent to the console (4, blue) to initiate the imaging sequence in (c). The signal is recorded on both the Bruker console (5) and on the UHFLI (3). For the reference images, an RF channel (yellow) is connected. c) EPI sequences: Compared to the standard Bruker sequence (1), the RASER‐EPI experiments (2) employ no RF excitation and no slice selection. The unaltered train of phase and readout gradients starts as soon as the signal generated by RASER action surpasses a set trigger threshold (blue).

All experiments were conducted on an 11.7 T (500 MHz ^1^H) Bruker AVANCE NEO spectrometer equipped with a microimaging three‐axis gradient system (micro5 probe) and a saddle coil. The RASER signals were passed through a low‐noise amplifier (ZX60‐P103LN+, Mini‐Circuits) and split using an RF splitter (Z99SC‐62‐S+, Mini‐Circuits), where one branch was connected to the Bruker spectrometer receiver, while the other one was attached to a Zurich Instruments UHFLI 600 MHz lock‐in amplifier, controlled by the LabOne toolset running on a separate computer, Figure 1b. The UHFLI's role is to digitally demodulate the RASER signals, and to continuously monitor their amplitude to trigger the Bruker EPI acquisition when exceeding a certain threshold. The data acquired on the Bruker receiver was processed using Paravision360 (see Supporting Information). In this way, we recorded 2D RASER images within 78 ms with a large matrix size of 128x128 pixels over the field of view (FOV) of 7 mm, with an in‐plane spatial resolution of 54x54 µm^2^ at a bandwidth of 468.75 kHz (3662 Hz/pixel) without slice selection.

Following this procedure, we acquired RASER images as depicted in Figure 2. The thermally polarized and SABRE HP reference images Figure 2a,b have the shape of the 5 mm NMR tube (ID: 4.1 mm) with slight distortions due to *B*_1_ inhomogeneity of our detection coil (see Supporting Information for the *B*_0_ map). The RASER image in Figure 2c has the same size and shape as the reference images acquired with conventional MRI of both the thermally polarized reference image (Figure 2a), and the hyperpolarized, non‐RASER reference image (Figure 2b). For the RASER image, the trigger threshold was chosen at 6 mV total signal, well below the typically‐reached 10 mV signal for these samples. By choosing the trigger threshold, the SNR for the entire image is set, giving an average SNR of 20.7 (details on SNR in Supporting Information).

**Figure 2:**
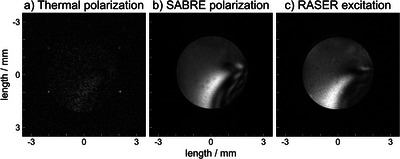
EPI images using a) thermal polarization b) SABRE polarization and c) RASER excitation. The images are recorded by EPI without slice selection resulting in a 2D image (5 mm NMR tube with 4.1 mm inner diameter, artefact in the bottom right due to distortion in *B*_0_ as shown in Supporting Information). The 128x128 images were acquired using 468.75 kHz bandwidth, 7x7 mm^2^ FOV, 273 µs echo time per line in k‐space, total image time 78.2 ms, and a shim of *T*_2_^*^  =  0.11 s. The sample consisted of 120 mM pyrazine and 6 mM [Ir(COD)(IMes)Cl] in d_4_‐methanol. The RASER image was recorded following the procedure in Figure 1 using 6 bar parahydrogen. The respective SNRs of the images are 1.5, 26.0, and 20.7. The polarization for the SABRE reference image was kept slightly below 1% to match the SNR of the RASER image.

The presented RASER MRI approach offers two remarkable advantages:

First, the utilization of the magnetization threshold ensures that a series of images can be recorded with the same level of MRI signal despite *T*_1_ decay using any concentration or polarization value, enabling more streamlined image reconstruction. A common challenge of HP MRI is the constantly decaying magnetization (and MRI signal by extension). Right after injection of the HP contrast agent, the product of concentration and polarization is high, giving a high magnetization which decays with *T*_1_. Compensating for *T*_1_ decay is critical when recording a series of scans for dynamic tracking, multi‐slice imaging or 3D acquisitions. Indeed, we demonstrate that the RASER MRI approach provides a series of images with identical signal level even under conditions of decaying hyperpolarization by setting the trigger voltage.

Second, the employed lock‐in amplifier enables direct MRI signal acquisition without the need to record the signal on the MRI scanner. As a result, the presented approach completely mitigates the need for entire transmit‐receive‐RF chain of MRI scanners to efficiently scan HP contrast media, which we demonstrate here for HP protons in pyrazine. We envision our approach to offer more advantages for ^13^C and ^129^Xe contrast media applications in the future, and believe that RASER MRI opens a new chapter for the emerging field of HP MRI.

To demonstrate the ability to measure consecutive images, a series of eight images was acquired (Figure 3). During image acquisition, the strong imaging gradients of 1.58 T/m bring the RASER below threshold and stop RASER action (for details on the RASER threshold, see Supporting Information). Spontaneous RASER build‐up of XY magnetization and image encoding (Figure 1a, steps 4 and 5) are repeated eight times (Figure 3a). After the images are acquired, the RASER can evolve “freely”, without disruption by gradients. In this way, leftover polarization is flipped into the transverse plane (marked free RASER burst in Figure 3a). The acquisition sequence was triggered at a threshold of 2 mV, yielding an average SNR of 14.0 for each image shown in Figure 3b. This approach is equivalent to a series of low‐flip‐angle pulses, often applied when using hyperpolarized markers. However, the amount of signal for each image remains exactly the same in this case, equivalent to flip‐angle sequences that gradually increase the flip angle to maintain a constant SNR.

**Figure 3:**
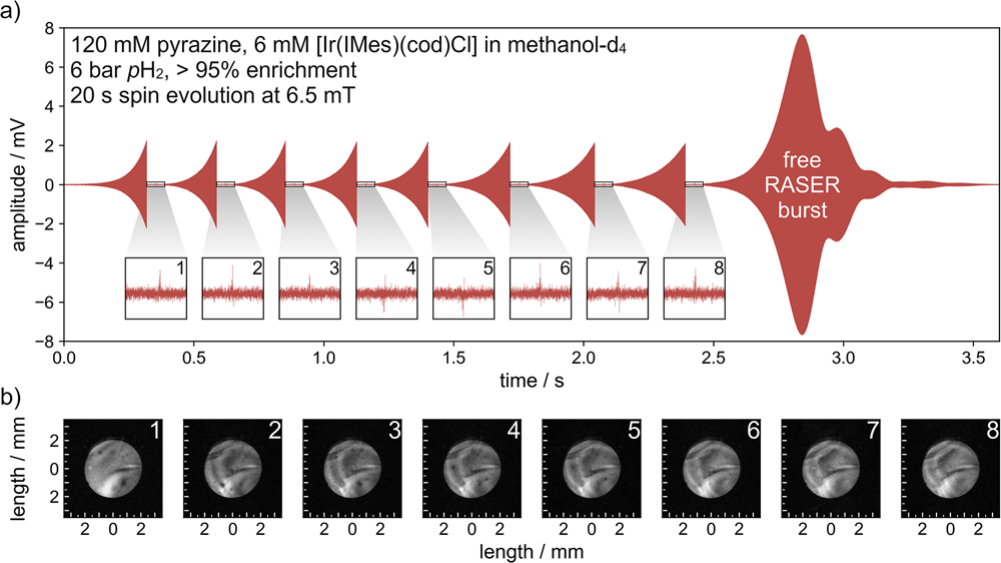
Eight consecutively recorded EPI images employing RASER excitation. a) Signal recorded on the Zurich Instruments lock‐in amplifier. b) RASER images 1–8 from Paravision 360 using the same experimental procedure and parameters as in Figure 2, except for the trigger threshold, which was kept three times lower yielding an SNR of 14.0 for these images. Some images show hydrogen bubbles (black dots).

In this study, we recorded rapid RASER MRI without the need for RF excitation. We employed SABRE hyperpolarization to obtain a population inversion of nuclear spin polarization, exploited the RASER dynamics for self‐excitation, and detected the MR images with conventional EPI encoding. In this way, we obtain a background‐free signal, only stemming from the (inverted) spins of interest.^[^ ^18^ ^]^ No other spins contribute to the signal because there is no RF pulse to excite them. In the present demonstration, the gradient strength is purposefully chosen so high as to suppress RASER activity during acquisition. This choice sacrifices the potential of RASER MRI for enhanced contrast,^[^ ^19^, ^20^, ^21^, ^22^ ^]^ but avoids artifacts caused by distant dipolar fields^[^ ^23^ ^]^ or other nonlinear effects,^[^ ^24^, ^25^, ^26^ ^]^ which can otherwise plague RASER images,^[^ ^21^ ^]^ and would have to be addressed, for example, by post‐processing using ML (Machine Learning).^[^ ^27^ ^]^

In the future, rapid RASER MRI could be applied to molecular contrast agents such as HP ^13^C pyruvate, which is FDA approved and part of over 20 clinical trials to track cancer metabolism, or HP ^129^Xe gas, which is already used for pulmonary imaging in patients and covered by health insurances since 2025. For the current clinical studies with these molecular contrast agents, dedicated ^13^C or ^129^Xe RF‐channels are required, which most clinical MRI scanners do not have. The RASER MRI approach circumvents the need for dedicated channels, as it only requires a receiver coil at the corresponding resonance frequency. Moreover, for both ^13^C and ^129^Xe, RASER activity has already been demonstrated.^[^ ^28^, ^29^ ^]^ The reported method solely relies on the detection coil, magnetic field, and gradients from the MRI system on which we demonstrated RASER MRI. The RF chain and acquisition hardware are provided by portable, simple‐to‐interface, commercially available instrumentation. Thus, we envision streamlined adaptation in clinical workflows, especially with hyperpolarized ^129^Xe, where specialized FDA‐approved MRI coils are already commercially available. The reduced hardware requirement is also ideal for miniaturized systems, while eliminating the need for RF pulses avoids excitation leakage in multi‐channel MR and may even pave the way for GHz MRI, as there is no tissue heating.

While the RASER threshold was readily met for this study, the clinically relevant conditions for more threshold‐demanding ^13^C and ^129^Xe can be achieved via Q‐boosting approaches, such as active feedback^[^ ^30^, ^31^ ^]^ or parametric pumping.^[^ ^32^ ^]^

## Conflict of Interests

EYC discloses a stake of ownership in XeUS Technologies LTD and Perxeus Technologies Inc. JGK is a shareholder of Voxalytic GmbH. The other authors declare no competing interests.

## Supporting information



Supporting Information

## Data Availability

The MRI data for this article is available at RADAR4KIT: https://doi.org/10.35097/kapcf985935rnu2g.
