# Supplementary material for: Rapid RASER MRI
Source: Angew Chem Int Ed Engl. 2026 Jan 28;65(10):e25699. doi: 10.1002/anie.202525699 (PMC12955514; doi:10.1002/anie.202525699)
Supplement: Supplementary file 1 — Supporting Information [file ANIE-65-e25699-s001.pdf]

Supplementary information

Angewandte Chemie

Rapid RASER MRI

Sören Lehmkuhl, Simon Fleischer, Jing Yang, Eduard Y. Chekmenev, Thomas Theis, Stephan Appelt, Jan G. Korvink, Mazin Jouda

Table of Contents

1. NMR sample preparation and SABRE hyperpolarization 3

2. The RASER threshold 3

3. Data acquisition and processing 4

4. B_0 field map of the 5 mm ^1H coil 4

5. SNR Estimation for Fig. 2 and Fig. 3 of the main text 5

6. References 5

1. NMR sample preparation and SABRE hyperpolarization

Hydrogen was cooled to 25 K in a closed-cycle cryostat setup (Advanced Research Systems), generating >98% enriched para-hydrogen, which was stored in transportable aluminum cylinders up to a pressure of 20 bar. The d_4-methanol used as solvent for the SABRE samples was acquired from Sigma Aldrich and degassed over four freeze-pump-thaw cycles. All subsequent manipulations were performed under argon atmosphere, using standard Schlenk techniques. The SABRE catalyst precursor [Ir(IMes)(COD)Cl]; (IMes = 1,3-bis(2,4,6-trimethylphenyl)imidazol-2-ylidene; COD = cyclooctadiene) was synthesized through previously published methods.[1] The chemicals for the synthesis as well as pyrazine were acquired from Sigma Aldrich and used as received. Final concentrations of the samples were 120 mmol/L of pyrazine and 6 mmol/L of catalyst precursor. Catalyst activation was initiated directly prior to each experiment, as the argon atmosphere within the high-pressure NMR-tube (Quick pressure valve tubes) was replaced by 4-5 bar parahydrogen.

2. The RASER threshold

Surpassing the RASER threshold is critical for any RASER experiment. The key parameters to surpass the threshold are good shims (T_2^*), a good resonator ($\eta \cdot Q$), and a high total population inversion ($-n_s P$) (details on surpassing the RASER threshold see below). Additionally, surface coils can alleviate the drawback of small filing factors in volume coils and provide a good quality factor in the region of interest.

An inversion (negative polarization) of the nuclear spin states can be achieved by RF-pulses[2], active feedback[3] or hyperpolarization techniques such as Dynamic Nuclear Polarization (DNP)[4], Spin Exchange Optical Pumping) SEOP[5] and Parahydrogen Induced Polarization (PHIP)[6]. Using RF-pulses requires high thermal polarization and is therefore mostly limited to highly concentrated samples. Additionally, it defeats the purpose of this study (working without RF-excitation). Active feedback[3] or parametric pumping[7] can increase the quality factor Q (and with that lower the RASER threshold) 1 million-fold, increasing the viability of the approach. This can even be combined with hyperpolarization techniques, which allow for large polarizations (over 1000-fold enhancement) on the hyperpolarized component.

A convenient way to describe a RASER system is defining a parameter ϵ, which describes how far the system is above the threshold. For example with $\epsilon = 3$ the system is a factor of three above the RASER threshold, while for $\epsilon < 1$ the system is below threshold and no RASER is observed. For a single RASER mode, this threshold parameter ϵ can be defined as the ratio of the transverse relaxation rate T_2^* and the radiation damping time constant τ_{rd}, giving

$$\epsilon = T_2^*/\tau_{rd}. \quad (\mathbf{1})$$

The transverse relaxation rate T_2^* can be increased by shimming to yield a magnetic field as homogenous as possible, but is ultimately limited by the inherent T_2 of a given sample. Additionally, any magnetic field gradient decreases T_2^*, and with that also decreases ϵ

The radiation damping time constant τ_{rd} of a given experiment in the context of the RASER is described in detail in Appelt *et al.*[8]. Following this work, it can be formulated (in SI units) as

$$1/\tau_{rd} = -1/4\ \mu_0\ \hbar\ \gamma^2\ \eta\ Q \cdot (n_S P). \quad (\mathbf{2})$$

In **Eq. 2**, μ_0 is the vacuum permeability, $\hbar$ Planck's constant, γ the nucleus-specific gyromagnetic ratio, η the coil filling factor, n_S the number of spins, P the polarization and Q the quality factor of the resonator.

Reaching the RASER threshold (achieving a high $\epsilon = T_2^*/\tau_{rd}$) is more challenging to meet in e.g. a clinical MRI scanner than in this study. Nonetheless, all the parameters can be adjusted and the RASER threshold drastically reduced.

3. Data acquisition and processing

The signal from the probe is split and detected on both the UHFLI used for triggering as well as the Bruker console (see Figure S1). On the console, the data is acquired using the Bruker Paravision360 software suite, version 3.2, using a modification of the standard EPI protocol. Specifically, both the RF excitation pulse and the slice selection gradient were removed. Images are acquired with 128x128 pixels, a field-of-view of 7x7 mm and a total bandwidth of 468.17 kHz. These settings result in a resolution of 54.7 µm and a per-pixel bandwidth of 3.66 kHz. Double sampling is employed, increasing the effective acquisition time from 38.6 ms to 78.2 ms. From the acquired data, the image is extracted as an 8-bit greyscale bitmap. A custom python script is used to calculate SNR after selecting equal areas of signal and noise.

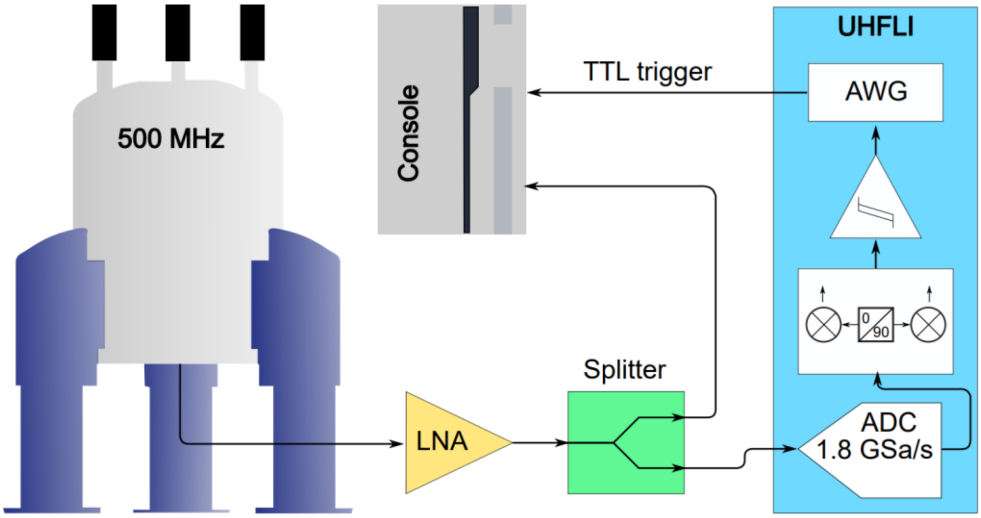

Figure S1. Signal path of the RASER MRI experiment. The MR signals from the probe are amplified using the ZX60-P103LN+ low-noise amplifier and then split using the Z99SC-62-S+ RF splitter. One branch of the signal is introduced to the Bruker console, while the other branch is connected to the UHFLI lock-in amplifier, where it is digitized at 1.8 GSa/s and then digitally demodulated to extract its magnitude and phase. Once the magnitude of the MR signal exceeds a certain threshold, an arbitrary waveform generator (AWG) module is activated to send a TTL signal to trigger the Bruker console to acquire data. The trigger delay was set to 1 ms for all experiments.

4. B_0 field map of the 5 mm ^1H coil

The images are distorted by our slightly damaged ^{1}H saddle coil. In Figure S2, we provide examples of axial slices in an H_2O/D_2O (1:10) phantom recorded at an isotropic resolution on 109 µm. The corresponding B_0 (and also B_1) field maps can be found in the ESI repository, together with the experimental data. These field maps consist of 183x64x64 pixels for both phase and magnitude provide a field-of-view of 20x7x7 mm.

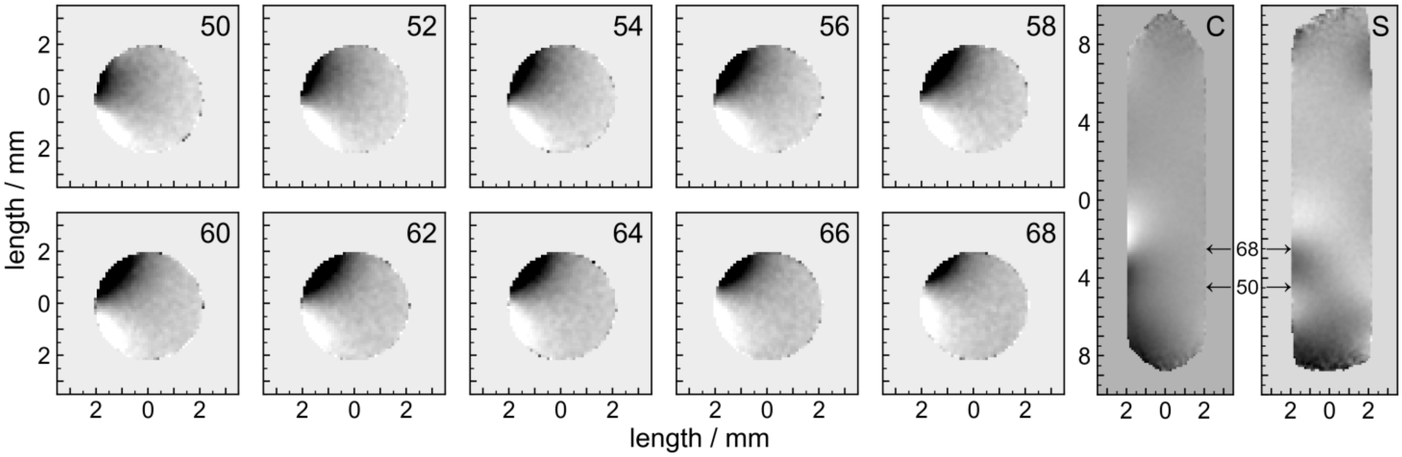

Figure S2. Axial slices number 50, 52, 54, 56, 58, 60, 62, 64, 66, and 68 of the B_0 map, as well as the mid-coronal (C) and mid-sagittal (S) slice. The slices show a distortion matching with the artifact in Figures 2 and 3 in the main text.

5. SNR Estimation for Fig. 2 and Fig. 3 of the main text

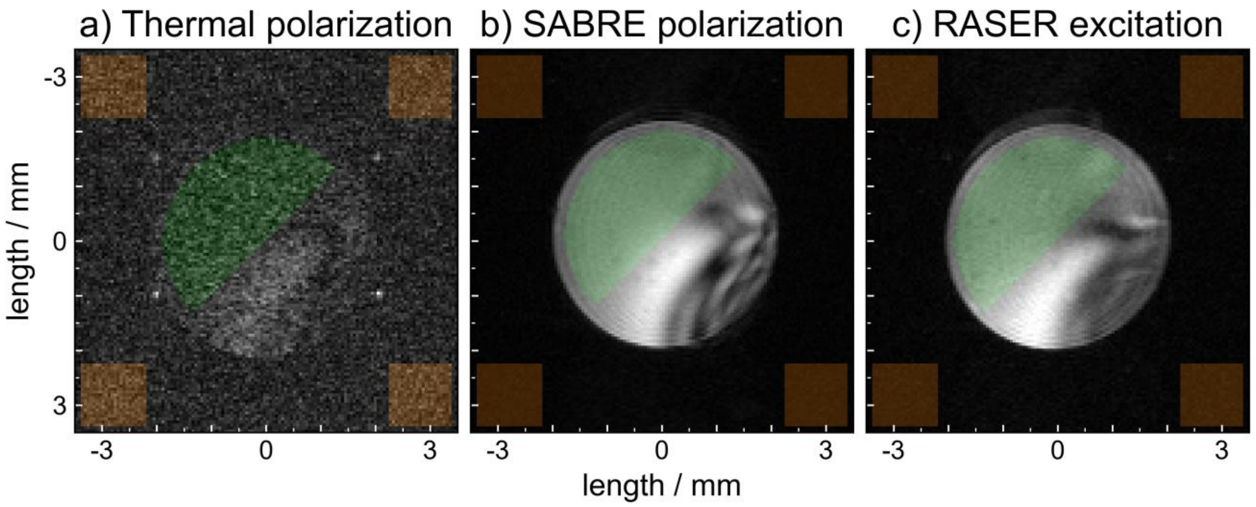

Figure S3. Estimating the average SNR in Fig.2: The chosen signal area is highlighted in green and the noise area in orange. Only part of the sample is considered to avoid artifact-plagued areas. a) Image using thermal polarization at 11.7T (500 MHz). b) Image using SABRE hyperpolarization with similar polarization as in (c). c) Image using RASER excitation. As describes in Fig.2, the images are recorded by EPI without slice selection resulting in a 2D image (5 mm NMR tube with 4.1 mm inner diameter). The 128x128 images were acquired using 469.75 kHz bandwidth, 7x7 mm FOV, 273 µs echo time per k-space line, repetition time 78.2 ms, and $T_2^* = 0.11$ s. The sample consisted of 120 mM pyrazine and 6 mM [Ir(COD)(IMes)Cl] in d4-methanol. The RASER image was recorded following the scheme in Fig. 2 and using 6 bar parahydrogen. The respective SNRs of the images are 1.5, 26.0 and 20.7. The polarization in the SABRE sample for the SABRE reference image was kept to below 1% to match the SNR of the RASER image.

Table S1. Average SNRs of Fig. 2 and Fig. 3

Experiment	SNR
Fig. 2a (Thermal polarization)	1.5
Fig. 2b (SABRE hyperpolarization)	26.0
Fig. 2c (RASER excitation, 6 mV trigger threshold)	20.7
Fig. 3 (RASER excitation, 2 mV trigger threshold)	14.0

6. References

[1] M. Becker, F. Arvidsson, J. Bertilson, E. Aslanikashvili, J. G. Korvink, M. Jouda, S. Lehmkuhl, *Magn Reson Imaging* **2025**, *115*, 110247.
[2] J. Schlagnitweit, S. W. Morgan, M. Nausner, N. Müller, H. Desvaux, *ChemPhysChem* **2012**, *13*, 482-487.
[3] V. Chacko, A. Louis-Joseph, D. Abergel, *Phys Rev Lett* **2024**, *133*, 158001.
[4] a) M. Abraham, M. A. H. Mccausland, F. N. H. Robinson, *Phys. Rev. Lett.* **1959**, *2*, 449-451; b) W. A. Barker, *Rev Mod Phys* **1962**, *34*, 173-&.
[5] a) G. Navon, Y. Q. Song, T. Room, S. Appelt, R. E. Taylor, A. Pines, *Science* **1996**, *271*, 1848-1851; b) T. G. Walker, W. Happer, *Rev Mod Phys* **1997**, *69*, 629-642.
[6] a) C. R. Bowers, D. P. Weitekamp, *Phys. Rev. Lett.* **1986**, *57*, 2645-2648; b) C. R. Bowers, D. P. Weitekamp, *J. Am. Chem. Soc.* **1987**, *109*, 5541-5542.
[7] I. Adelabu, S. Nantogma, S. Fleischer, M. Abdulmojeed, H. de Maissin, A. B. Schmidt, S. Lehmkuhl, M. S. Rosen, S. Appelt, T. Theis, C. Qian, E. Y. Chekmenev, *Angewandte Chemie* **2024**, e202406551.
[8] S. Appelt, A. Kentner, S. Lehmkuhl, B. Blümich, *Prog. Nucl. Magn. Reson. Spectrosc.* **2019**, *114-115*, 1-32.
